# A 2D video-analysis scoring system of 90° change of direction technique identifies football players with high knee abduction moment

**DOI:** 10.1007/s00167-021-06571-2

**Published:** 2021-04-29

**Authors:** Francesco Della Villa, Stefano Di Paolo, Dario Santagati, Edoardo Della Croce, Nicola Francesco Lopomo, Alberto Grassi, Stefano Zaffagnini

**Affiliations:** 1 Education and Research Department, Isokinetic Medical Group, FIFA Medical Centre of Excellence, Bologna, Italy; 2 Department for Life Quality Studies QUVI, Università Di Bologna, Via Giulio Cesare Pupilli, 1, 40136 Bologna, BO Italy; 3 Department of Information Engineering, University of Brescia, Brescia, Italy; 4 2nd Orthopaedic and Traumatologic Clinic, IRCCS Istituto Ortopedico Rizzoli, Bologna, Italy; 5 Department of Biomedical and Neuromotor Sciences, University of Bologna, Bologna, Italy

**Keywords:** ACL, Return to sport, 2D video analysis, ACL injury prevention, Cut maneuver

## Abstract

**Purpose:**

Abnormal joint biomechanics and poor neuromuscular control are modifiable risk factors for Anterior Cruciate Ligament (ACL) injury. Although 3D motion capture is the gold standard for the biomechanical evaluation of high-speed multidirectional movements, 2D video analysis is a growing-interest alternative because of its higher cost-effectiveness and interpretability. The aim of the present study was to explore the possible association between a 2D evaluation of a 90° change of direction (COD) and the KAM measured with gold standard 3D motion analysis.

**Methods:**

Thirty-four competitive football (soccer) players (age 22.8 ± 4.1, 18 male and 16 females) were enrolled. Each athlete performed a series of pre-planned 90° COD at the maximum speed possible in a laboratory equipped with artificial turf. 3D motion analysis was recorded using 10 stereophotogrammetric cameras, a force platform, and three high-speed cameras. The 2D evaluation was performed through a scoring system based on the video analysis of frontal and sagittal plane joint kinematics. Five scoring criteria were adopted: limb stability (LS), pelvis stability (PS), trunk stability (TS), shock absorption (SA), and movement strategy (MS). For each criterion, a sub-score of 0/2 (non-adequate), 1/2 (partially adequate), or 2/2 (adequate) was attributed to the movement, based on objective measurements. The intra-rater and inter-rater reliability were calculated for each criterion and the total score. The Knee Abduction Moment (KAM) was extracted from the 3D motion analysis and grouped according to the results of the 2D evaluation.

**Results:**

Excellent intra-rater reliability (ICC > 0.88) and good-to-excellent inter-rater reliability (ICC 0.68–0.92) were found. Significantly higher KAM was found for athletes obtaining a 0/2 score compared to those obtaining a 2/2 score in all the sub-criteria and the total score (20–47% higher, *p* < 0.05). The total score and the LS score showed the best discriminative power between the three groups.

**Conclusion:**

The 2D video-analysis scoring system here described was a simple and effective tool to discriminate athletes with high and low KAM in the assessment of a 90° COD and could be a potential method to identify athletes at high risk of non-contact ACL injury.

**Level of evidence:**

IV.

**Supplementary Information:**

The online version contains supplementary material available at 10.1007/s00167-021-06571-2.

## Introduction

Anterior Cruciate Ligament (ACL) injuries are a real challenge for sports medicine practice. Return to sport (RTS) at the pre-injury level is not guaranteed, and the re-injury rate is still high [39], up to 20–30% in young and active patients [37].

Several primary prevention protocols based on neuromuscular training (NMT) have been proposed [13, 25, 36]. Attention has been paid to identifying modifiable risk factors through a general or targeted neuromuscular intervention [3]. Among these risk factors, frequently mentioned and discussed topics are abnormal joint biomechanics and poor neuromuscular control [20–22]. Identifying high-risk individuals may allow personalizing the preventative intervention, targeting added NMT training to the high-risk population [12, 33].

Poor lower limb frontal plane control is a modifiable factor associated with a higher risk of injury [26, 28, 40], such as ACL injuries. Inadequate ability to control knee movements on the frontal plane can manifest as high dynamic knee valgus (DKV) loading, described as altered hip and knee kinematics observed in the frontal and transverse planes during weight-bearing activities [7, 10, 40]. Knee Abduction Moment (KAM) is widely recognized as one of the main indicators for ACL injury risk [16, 26], as an element of DKV loading. Hewett et al. demonstrated that female athletes suffering an ACL injury during competition showed an altered neuromuscular control with 2.5 times greater KAM during the drop vertical jump (DVJ) task than non-injured athletes [14]. In a similar study, Krosshaug et al. challenged this theory, finding no difference in KAM but an effect on medial knee motion on ACL injury risk [20]. Studies with prospective design evaluating ACL injury risk in movements different from DVJ are lacking. Identifying abnormal KAM in high-speed athletic tasks may be useful for clinicians to predict the ACL injury risk and target more primary and secondary preventative training [34].

A 3D marker-based video analysis system is costly in terms of advanced technical skills, data analysis, and processing time, thus not always applicable to ACL prevention and rehabilitation’s daily clinical practice. The 2D video analysis approach is cost-effective, user-friendly, and reliable to screen excessive valgus [23, 29, 40]. Several tests have been proposed to estimate DKV loading in jumping tasks [4, 17, 30], while only two study groups validated a score on cutting techniques based on 2D video analysis vs 3D motion capture [11, 38]. Testing high load mono-podalic movements may be useful as more reproductive of the natural playing scenario, to identify athletes and patients with abnormal control of the lower limb. Such evaluations may serve as screening tools to target specific interventions to reduce the DKV loading.

Therefore, the aim of the present study was to explore the possible association between 2D evaluation of a 90° change of direction and the KAM measured with gold standard 3D motion analysis. The hypothesis was that poorer 2D evaluation would have correlated to higher KAM.

## Materials and methods

The study was approved by the Institutional Review Board (IRB approval: 555/2018/Sper/IOR of 12/09/2018) of Area Vasta Emilia Romagna Centro (AVEC, Bologna, Italy) and registered on ClinicalTrials.gov (Identifier: NCT03840551). All the subjects signed informed consent before starting the acquisition protocol.

### Participants

The analysis was conducted in the Education and Research Department of the Isokinetic Medical Center of Bologna (Italy). Overall, 34 recreational and elite footballers were recruited for the study (Table 1). Inclusion criteria were age between 18 and 50 years and Tegner activity level at least 7. Exclusion criteria were: (1) evidence of musculoskeletal disorders or functional impairment; (2) body mass index (BMI) > 35; (3) previous surgery to lower limbs; (4) cardiopulmonary or cardiovascular disorders; (5) inability to perform the required tasks.

**Table 1:** Demographic data of the subjects enrolled in the study

Demographic data	
Number of subjects	34
Age	22.8 ± 4.1 [18–31]
Gender (m/f)	18/16
Height (cm)	174.8 ± 10.2 [157–191]
Weight (kg)	68.6 ± 12.7 [51–94]
BMI	22.6 ± 2.6 [18–27]
Dominant limb (r/l)	30/4
Tegner	8.6 ± 1.0 [7–9]

Data were expressed as mean ± standard deviation [range]. Dominant limb is meant as the preferred one used to kick a ball

### COD acquisition protocol

**Fig. 1:**
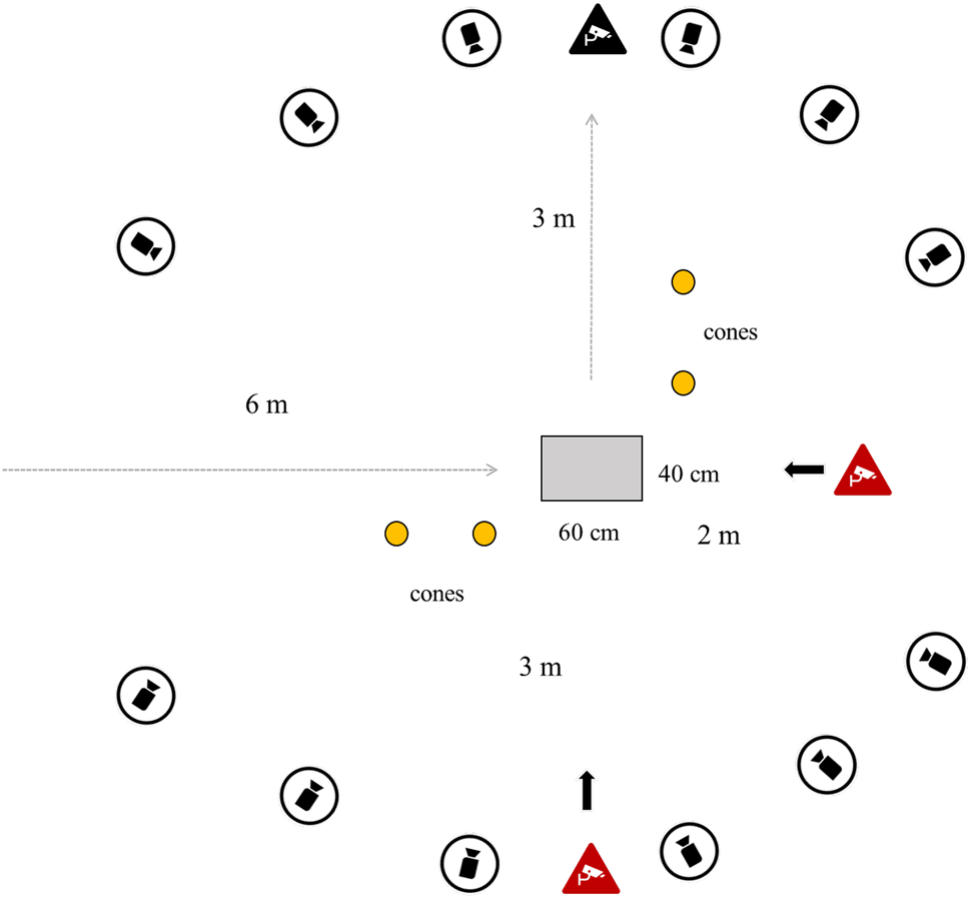
Laboratory setup. Gray cameras were used for the 3D motion capture of the KAM, while cameras in the triangle boxes were used for the 2D analysis. In the example figure, the 90° COD was performed with the right foot, thus one of the two lateral cameras (in the black triangle box) was not used for the 2D analysis (vice versa for a COD performed with left foot)

As part of a multi-movement assessment, each athlete was asked to perform a pre-planned 90° change of direction (COD) consisting of a frontal sprint followed by a 90° sidestep cut and a further frontal sprint in the new direction. The acquisition setting is reported in Fig. 1. Athletes were asked to complete the movements at the maximum speed possible (100%). Before the test, the subjects performed a 10-min dynamic warm-up and few repetitions of the movement to get confident with the environment and the motor task. A sport and exercise medicine physician specialized in sports biomechanics (FDV) instructed each subject on the movements to perform and verified each trial's validity. Full foot contact on the force platform was required to consider a trial valid. All subjects performed three valid repetitions per lower limb.

3D motion analysis was recorded through a set of 10 stereophotogrammetric cameras, a force platform embedded in the floor (AMTI 400*600, Watertown, MA USA), and three high-speed cameras placed frontally and bilaterally towards movement direction (VICON Nexus, Vicon Motion Systems Ltd, Oxford, UK) (Fig. 1). The sampling frequency of cameras and force platform was 120 Hz, while the sampling frequency of the high-speed cameras was 100 Hz. The systems were synchronized for direct data comparison. The laboratory floor was equipped with artificial turf.

The system calibration was performed at the beginning of the acquisition and repeated periodically during the session. A total of 42 retroreflective markers were placed on each subject according to the full-body Plug-in Gait protocol. The same expert user conducted the entire marker positioning process. After marker positioning, subjects’ model calibration was performed before each acquisition.

### Data processing—3D analysis

Regarding the 3D analysis, VICON Nexus was used to quantify the KAM. Marker trajectories were collected through the stereophotogrammetric cameras, and ground reaction force (GRF) were collected through the force platform. The KAM was quantified using the standard “bottom-up” inverse dynamics approach of the Plug-in Gait protocol. The entire phase of foot contact on the force platform was considered in the analysis. The peak KAM value was extracted for each trial and normalized by the subject's body weight (BW).

### Data processing—2D analysis

Regarding the 2D analysis, a scoring system was adopted based on the frontal and sagittal plane joint kinematics. Such scoring system is included in a clinical multiple movements evaluation for RTS decision making after ACL reconstruction [5, 6]. The test, a qualitative movement evaluation, is aimed to identify biomechanical and neuromuscular control deficits providing an intuitive and quick response to the patient. The evaluation is performed in a specific VICON software environment through the recordings of the three high-speed cameras and the resultant GRF vector of the force platform. Joint kinematics are evaluated at the frame of maximal knee flexion angle after the foot contact with the force platform.

**Fig. 2:**
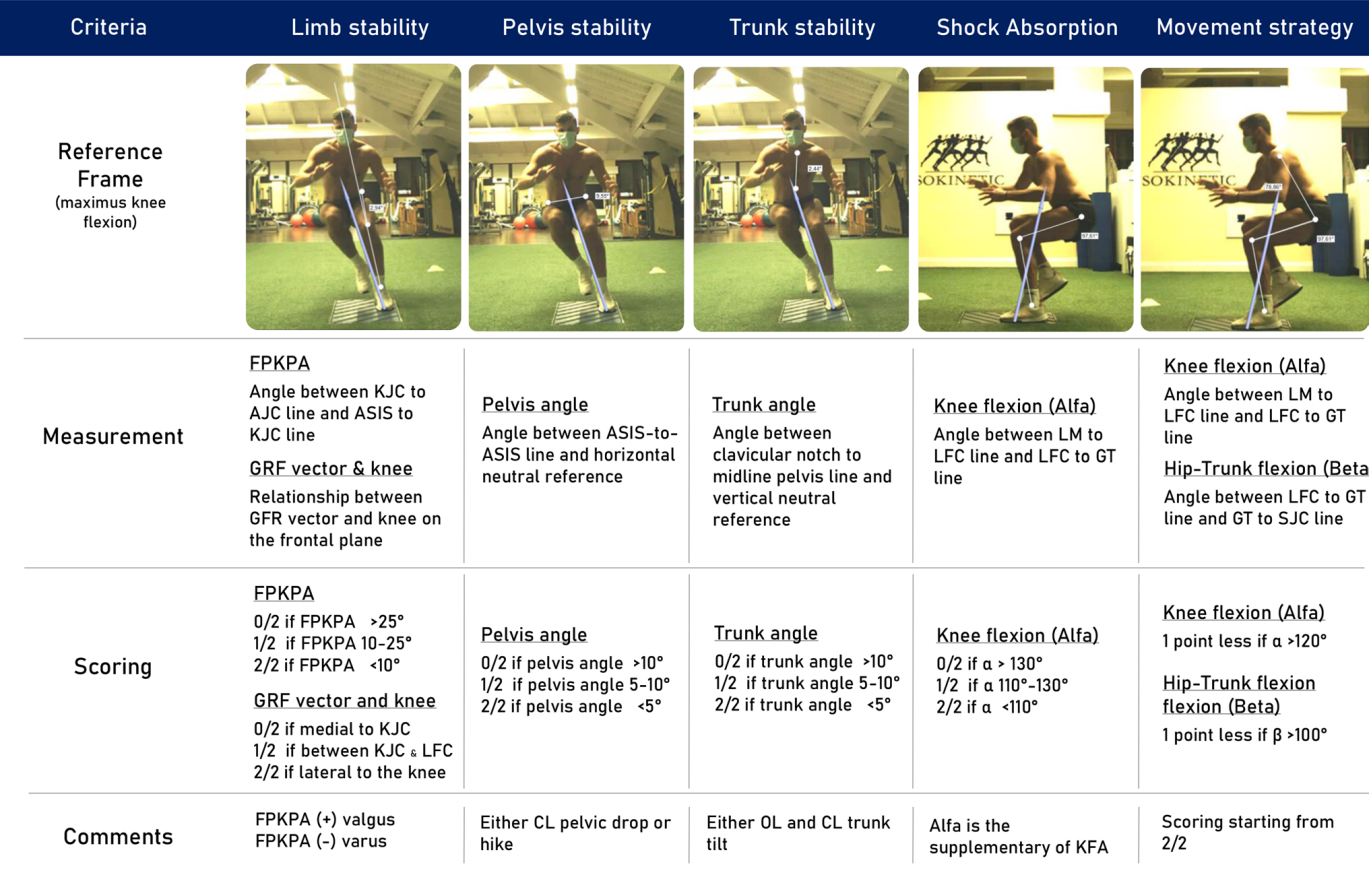
Detailed scoring system for 2D video analysis of COD. *FPKPA* frontal plane knee projection angle, *GRF* ground reaction forces, *KJC* knee joint center, *AJC* ankle joint center, *ASIS* antero-superior iliac spine, *LM* lateral malleolus, *LFC* lateral femoral condyle, *GT* great trochanter, *SJC* shoulder joint center, *CL* contralateral, *OL* omolateral, *KFA* knee flexion angle

For the present study, each COD trial was evaluated through five scoring criteria (modified from a scoring system developed by Prof. Christopher Powers at University of Southern California), *limb stability* (LS), *pelvis stability* (PS), *trunk stability* (TS), *shock absorption* (SA), and *movement strategy* (MS). For each criterion, a sub-score of *0/2* (non-adequate), *1/2* (partially adequate), or *2/2* (adequate) is attributed to the movement, based on objective measurements detailed in Fig. 2. A single sports physician specialized in sports biomechanics (FDV) evaluated each COD trial. The maximum total score for each trial is 10/10.

### Statistical analysis

The intraclass correlation coefficient (ICC) was used to calculate intra-rater and inter-rater reliability for each criterion and the total score. Reliability was considered poor, moderate, good, and excellent for ICC values lower than 0.50, between 0.50 and 0.75, between 0.75 and 0.90, and greater than 0.90, respectively [19].

The KAM extracted from each trial was grouped according to the results of the 2D evaluation. Five group distinctions were performed based on different 2D parameters, and three groups per distinction were generated. The groups were divided in terms of: LS (Groups 0, 1, 2); frontal plane knee projection angle—FPKPA (Groups < 25°, 25–40°, > 40°); ground reaction force—GRF vector score (Groups 0, 1, 2); TS (Groups 0, 1, 2); total score (Groups 0–4, 5–7, 8–10). The normal distribution of the data was verified through the Kolmogorov–Smirnov test. The categorical variables were presented as percentages over the total, while the normally distributed variables were presented as mean ± standard deviation. The ANOVA was used to investigate the statistical differences among the three groups, and the two-tails Student’s *t* test with Dunn–Sidak adjustment for multiple comparisons was used to investigate the differences between the single groups.

Furthermore, the Pearson’s coefficient *r* was used to investigate the linear correlation between the KAM and the FPKPA.

Differences were considered statistically significant for *p* < 0.05. All the statistical analyses were performed in MATLAB (The MathWorks, Natick, United States).

An a-priori power analysis was performed based on a previous similar study analyzing a 45° sidestep cut maneuver [35]. Considering a standard deviation of 0.4 N*m/BW (Newton-Meters/body weight) and a minimum effect size of 2.0, at least 14 subjects were required to have a power of 0.9.

## Results

Overall, 180 valid trials were included in the analysis. The average speed of the trials was 4.0 ± 0.3 m/s. The average peak KAM was 2.7 ± 1.0 N*m/BW and 2.5 ± 1.5 N*m/BW, respectively, for male and female subjects (n.s.).

For the sub-scores, the intra-rater reliability ranged from 0.88 to 1.00, while the inter-rater reliability ranged from 0.68 to 0.92. The total score showed an intra-rater and inter-rater reliability of 0.94 and 0.83, respectively.

**Fig. 3:**
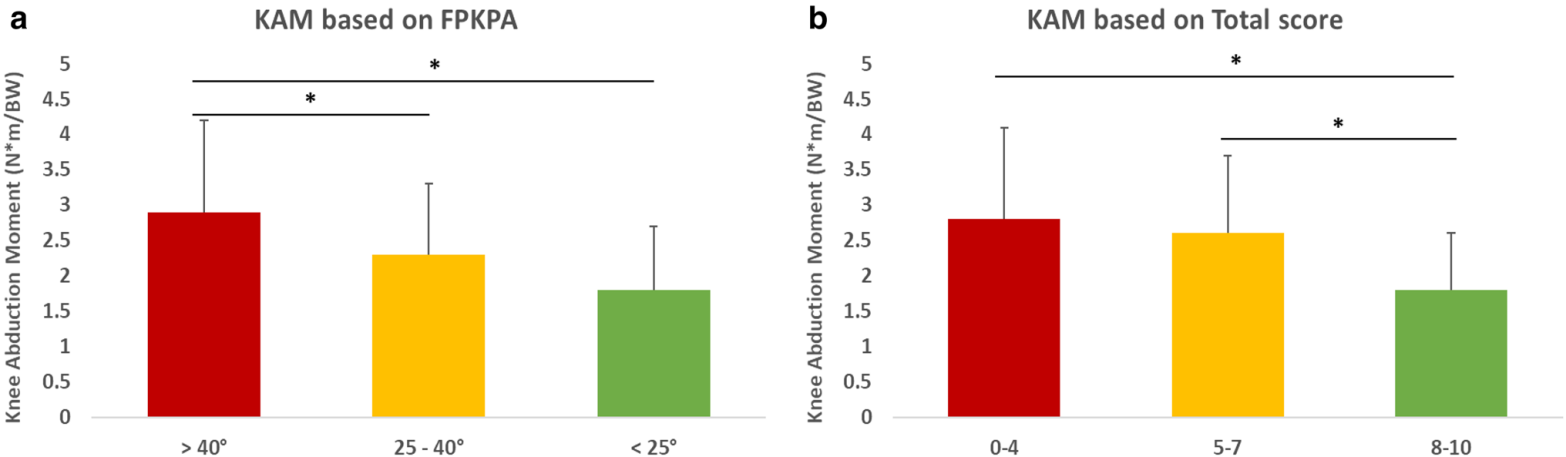
Knee abduction moment (KAM) based on **a** the frontal plane knee projection angle (FPKPA) (> 40°, 25–40°, < 25°) and **b** on the total score (0–4, 5–7, 8–10). Bars with asterisk represent statistically significant differences between the single groups (*p* < 0.05)

A statistically significant difference (*p* < 0.05) was found in terms of KAM among the three groups based on the LS score, the FPKPA, the GRF vector, and the total score (Table 2). In the LS score, in the trials score as 0, the KAM was 47% higher than the trials score as 2 (Table 3, Online Appendix A). The KAM associated with FPKPA higher than 40° was significantly higher than the other two groups (Fig. 3a). Furthermore, a statistically significant linear correlation was found between the KAM and FPKPA (*r* = 0.35, *p* < 0.0001). The KAM associated with a GRF vector score of 0 was significantly higher than the other two groups (online Appendix A). The KAM associated with total score 0–4 and 5–7 were significantly higher than total score 8–10 (Fig. 3b). In the TS score, the KAM was 20% higher in the trials scored as 0 compared to the trials score as 2 (Online Appendix A). The inverse bimodal distribution of the KAM over the trunk angle used for the TS score classification can be found in Online Appendix B.

**Table 2:** Knee Abduction Moment (KAM, [N*m/BW]) based on the different 2D evaluations used in the MAT test

2D evaluation	Groups			*p* value
Knee abduction moment based on the different 2D evaluations				
LS score	0	1	2	
	2.7 ± 1.2	2.3 ± 1.2	1.5 ± 0.4	0.0027*
FPKPA	> 40°	25–40°	< 25°	
	2.9 ± 1.3	2.3 ± 1	1.8 ± 0.9	< 0.0001*
GRF vector	0	1	2	
	2.8 ± 1.3	2.1 ± 0.8	1.9 ± 0.9	0.0047*
TS score	0	1	2	
	2.9 ± 1.4	2.6 ± 1.2	2.3 ± 1.0	n.s.
Total score	0–4	5–7	8–10	
	2.8 ± 1.2	2.6 ± 1.3	1.8 ± 0.8	0.0054*

Data are expressed as mean ± standard deviation. The asterisks represent statistically significant differences between the three groups evaluated through the ANOVA (*p* < 0.05)

*n.s.* non-significant

**Table 3:** Multiple comparisons of the Knee Abduction Moment (KAM, [N*m/BW]) based on the different 2D evaluations used in the MAT test

2D evaluation	Diff (%)	*p* value
Multiple comparisons for the different 2D evaluations		
LS score		
0 vs 1	0.5 (17%)	n.s.
0 vs 2	1.3 (47%)	< 0.0001*
1 vs 2	0.8 (35%)	n.s.
FPKPA		
> 40° vs 25–40°	0.6 (21%)	0.0023*
> 40° vs < 25°	1.1 (38%)	< 0.0001*
25–40° vs < 25°	0.5 (22%)	n.s.
GRF vector		
0 vs 1	0.7 (24%)	0.0147*
0 vs 2	0.9 (31%)	0.0012*
1 vs 2	0.2 (9%)	n.s.
TS score		
0 vs 1	0.3 (9%)	n.s
0 vs 2	0.6 (20%)	0.0115*
1 vs 2	0.3 (12%)	n.s.
Total score		
0–4 vs 5–7	0.2 (9%)	n.s.
0–4 vs 8–10	1 (37%)	0.0001*
5–7 vs 8–10	0.8 (31%)	0.0052*

Data are expressed as mean ± standard deviation. The asterisks represent statistically significant differences between single groups evaluated through the *t* test with Dunn–Sidak adjustment (*p* < 0.05)

*n.s.* non-significant

## Discussion

The main finding of the present study was a strong association for KAM between the gold standard 3D motion capture and a 2D video-analysis tool in the evaluation of 90° COD biomechanics. Excellent intra-rater and inter-rater reliability were also found for the 2D criteria and the total score. Therefore, the 2D evaluation here described could be a potential method to identify athletes at high risk for primary and secondary ACL injury in a simple, reliable, and cost-effective fashion.

Overall, in the evaluation of the present 2D scoring system of 90° COD, the lower the total score and the higher the magnitude of KAM. Moreover, each of the 2D tested criteria (LS score, FPKPA, GRF vector, TS score) allowed a clear distinction between the athletes performing good quality movements, i.e., *2/2* (lower KAM), and the athletes performing non-adequate movements, i.e., *0/2* (higher KAM).

A simple and effective tool to discriminate between athletes with acceptable (low KAM) and poor biomechanics (high KAM) may be valuable in targeting primary prevention or supporting the RTS decision making following non-contact knee injuries. Athletes’ defined as “at-risk” could indeed benefit from customized preventative programs and potentially reduce injury and re-injury risk. It has been demonstrated that athletes displaying higher KAM at jumping tasks benefit at a greater extent of targeted NMT [13]. However, there are different opinions on targeting additional preventative measures (e.g., for ACL injuries) in high-risk individuals, with researchers that challenged the application of screening test to stratify the injury risk [2]. Such an approach is less questioned regarding secondary prevention of ACL injuries, especially in young and active patients [31]. Qualitative movement assessment, alongside quantity (strength, hop tests), is warranted following ACL reconstruction, both in the pediatric and adult population [1, 9, 24]. The COD scoring system described here can also be applied in this second context as a criterion to RTS.

The group of athletes with a total score ranging from 8/10 to 10/10 (*high-quality COD*) showed significantly lower KAM (one third) than the other two groups. A high total score could be indicative of very limited risk of knee joint overloads and serve as a potential green light for the RTS. The total score also showed excellent intra-rater and good inter-rater reliability (ICC = 0.94 and 0.83, respectively), thus describing a robust overall measure of an athlete’s movement quality.

**Fig. 4:**
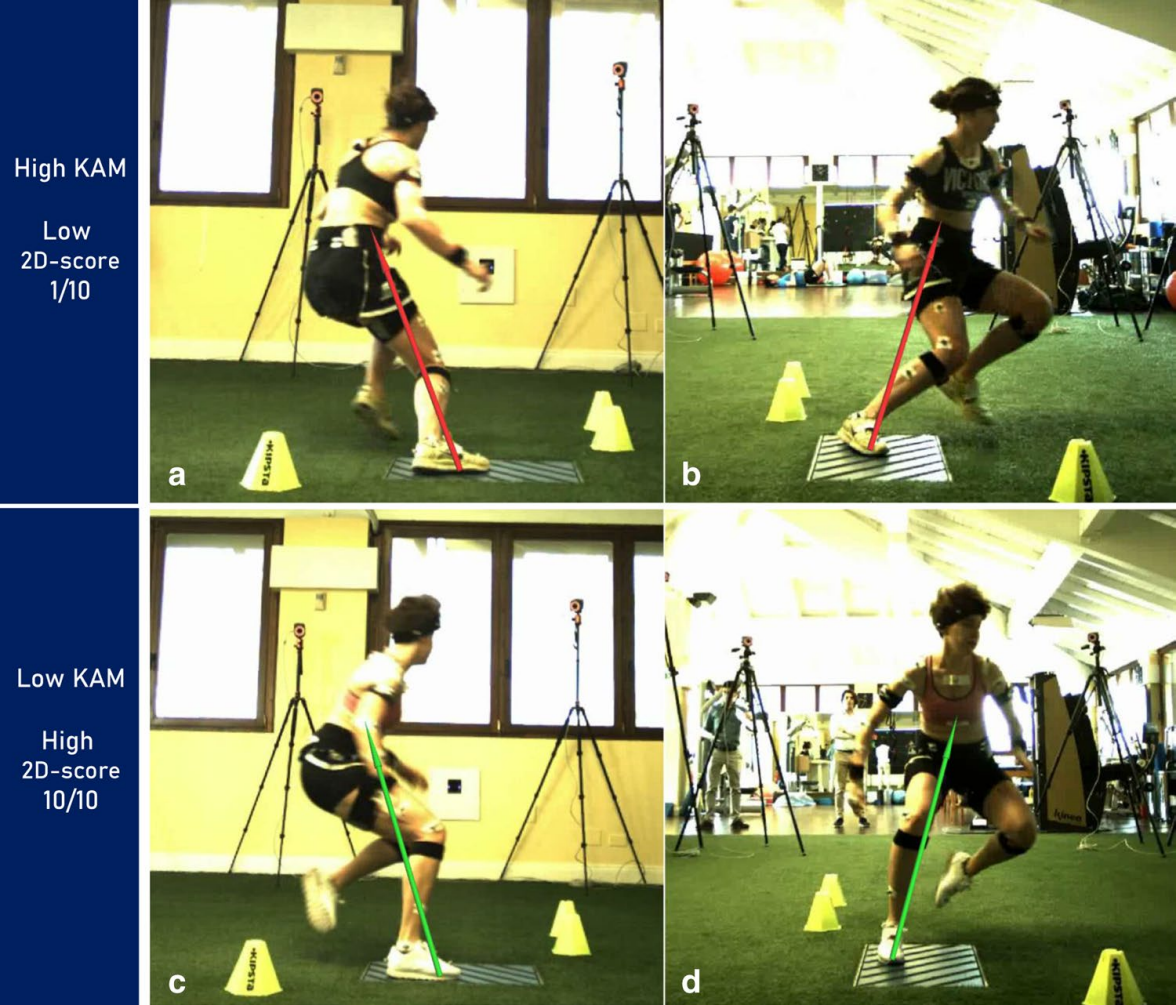
Example of movement (**a** lateral plane, **b** frontal plane) performed with high KAM and relative low limb stability and total score (top); example of movement (**c** lateral plane, **d** frontal plane) performed with low KAM and relative high limb stability and total score (bottom)

The sub-scoring defining the LS (evaluation of FPKPA and GRF vector at the KJC) showed the best discriminative power with significant differences between the three groups of athletes. A significant positive correlation between FPKPA and KAM was also found, like in previous COD biomechanical studies [38]. The sensitivity of the FPKPA and other measures of DKV as a screening tool for lower limb injury risk during different movements has been questioned in previous studies [3, 28, 32]. The present study confirmed the validity of FPKPA within a screening protocol for a multidirectional high-demanding task as the COD. Furthermore, the presence of an “external” GRF vector (lateral to the knee—Figs. 2 and 4) was significantly associated with higher KAM. It should be underlined that the GRF vector score was based on vector direction rather than magnitude. This last aspect hugely simplifies the use and the interpretation of such a score, making it suitable for easy and quick feedback to the athlete under assessment.

The concurrency of the criteria included in the 2D video-analysis screening tool described could effectively detect differences in KAM magnitude—and athletes’ ACL injury risk. The strength and novelty of this tool are the simple fashion for raters and the possibility to generate quick and comprehensible reports for the athletes.

Unlike the current literature, which mostly focused on the 2D assessment of jump landings, the present research focused on a 90° sidestep cut maneuver, broadly identified as the major high-risk movement during soccer [8, 10, 18]. Only two study groups previously validated a 2D video analysis tool against a gold standard 3D motion capture for the assessment of the cut maneuver [11, 38]. Both groups developed elegant and robust screening tools for ACL injury risk assessment in a laboratory environment. The present study results are in accordance with those of both groups regarding reliability and high-risk biomechanics detection. Slight methodological differences are present between the present and the cited studies. Compared to Weir et al., a lower number of parameters—namely, measurement methods—were used [38]. Compared to Dos’Santos et al., less technical features—namely, a third high-speed camera at 45° and a second force platform for penultimate foot contact evaluation—were required [11]. Such aspects could increase the effort and the time needed for the screening while limiting the interaction with the athletes under assessment.

Further inferences can be drawn from the results of the present study. Regarding the TS score, it was found that either contralateral or omolateral, the higher the trunk tilt, the higher the KAM (Online Appendix B). Therefore, the maintenance of a Neutral trunk position indicates limited risk for external knee joint moment occurrence. Such a trend is partially in contrast with the previous literature [15], which identified only omolateral trunk tilt as a risk factor for ACL injury. A possible explanation of such a difference can be found in trunk rotation. The presence of trunk rotation limits the visibility of anatomical references on either frontal or lateral view and could “cover” the presence of contralateral trunk tilt. Not surprisingly, trunk and pelvis criteria showed the lowest inter-rater reliability, as also underlined in previous studies [11].

Moreover, although a very selected healthy sportive population was investigated, low scores were detected for most athletes (about 60%), with no differences between men and women. Dos’Santos et al. also identified that only 33% of athletes under investigation reached high scores (low KAM) in sidestep cut maneuver [11]. This aspect might lead the sports practitioners to consider inserting a routine assessment of athletes’ biomechanics to detect risky situations and potentially reduce the teams’ injury rate.

The present study has some limitations. First, no muscle activation data were collected. Such data could have provided further valuable information towards injury risk assessment and corroborated the 2D scoring tool. Second, the lowest inter-rater agreement was found for the TS score. This could be due to the difficult identification of the midline pelvis and the clavicular notch in the athletes adopting trunk pre-rotation towards the movement direction in the reference frame. Third, the data collection was performed in one single session. A repeated evaluation of athletes’ biomechanics after a preventative training program could have highlighted interesting differences in the 3D evaluation as well as the sensibility of the 2D video-analysis. Lastly, the task evaluated in the present study was an anticipated change of direction. Further studies could be focused on the assessment of an unanticipated change of direction, both in terms of 3D and 2D evaluations.

The clinical relevance of the present work is that the implementation of the proposed 2D scoring system can help identify (uninjured) football players displaying excessive knee external loads during planned high-risk movements for ACL injury. Once a pre-participation screening is done, athletes with higher KAM (higher dynamic knee valgus loading) may benefit from additional preventative NMT. It is demonstrated that the athletes with poor biomechanics and a risky profile benefit more from targeted NMT [13, 27]. Moreover, once validated, there is room for further studying this kind of evaluation in secondary prevention following ACLR before RTP.

## Conclusion

The 2D video-analysis scoring system described in the present study was an effective tool to discriminate athletes with high and low KAM in the assessment of a 90° COD. Such a system could be a quick and cost-effective method to identify athletes at high risk of non-contact ACL injury and support orthopedic surgeons and sports physicians in RTS decision making.

## Supplementary Information

Below is the link to the electronic supplementary material.

Supplementary file1 (DOCX 134 kb)

Supplementary file2 (DOCX 64 kb)

## Abbreviations

ACL: Anterior cruciate ligament
BW: Body weight
BMI: Body mass index
COD: Change of direction
DVJ: Drop vertical jump
DKV: Dynamic knee valgus
FPKPA: Frontal plane knee projection angle
GRF: Ground reaction force
ICC: Intraclass correlation coefficient
KAM: Knee abduction moment
LS: Limb stability
MS: Movement strategy
NMT: Neuromuscular training
PS: Pelvis stability
RTS: Return to sport
SA: Shock absorption
TS: Trunk stability
