# Supplementary material for: A 2D video-analysis scoring system of 90° change of direction technique identifies football players with high knee abduction moment
Source: Knee Surg Sports Traumatol Arthrosc. 2021 Apr 29;30(11):3616–25. doi: 10.1007/s00167-021-06571-2 (PMC9568485; doi:10.1007/s00167-021-06571-2)
Supplement: Supplementary file 1 — Supplementary file1 (DOCX 134 kb) [file 167_2021_6571_MOESM1_ESM.docx]

**Appendix A**


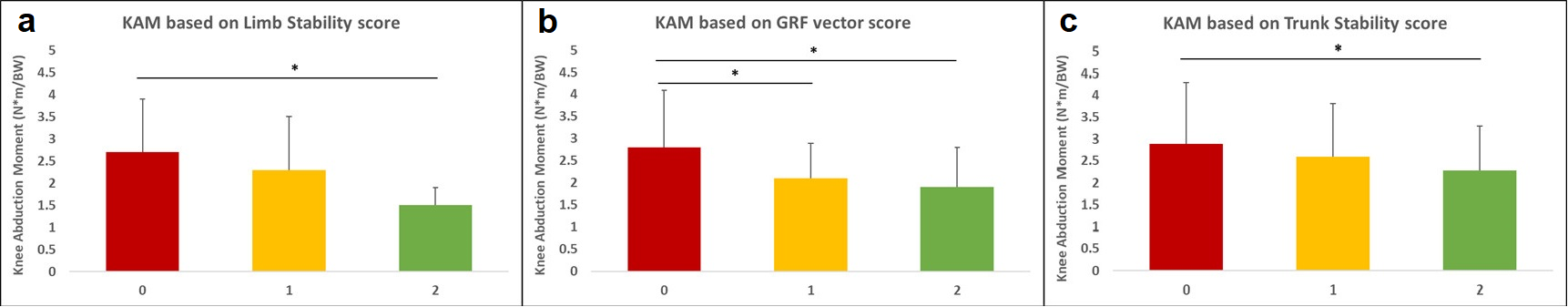


*Knee Abduction Moment (KAM) based on the Limb Stability score (a), the GRF vector score (b), the Trunk Stability score (c) (0, 1, 2). Bars with asterisk represent statistically significant differences between the single groups (p<0.05).*
