# Supplementary material for: A 2D video-analysis scoring system of 90° change of direction technique identifies football players with high knee abduction moment
Source: Knee Surg Sports Traumatol Arthrosc. 2021 Apr 29;30(11):3616–25. doi: 10.1007/s00167-021-06571-2 (PMC9568485; doi:10.1007/s00167-021-06571-2)
Supplement: Supplementary file 2 — Supplementary file2 (DOCX 64 kb) [file 167_2021_6571_MOESM2_ESM.docx]

**Appendix B**


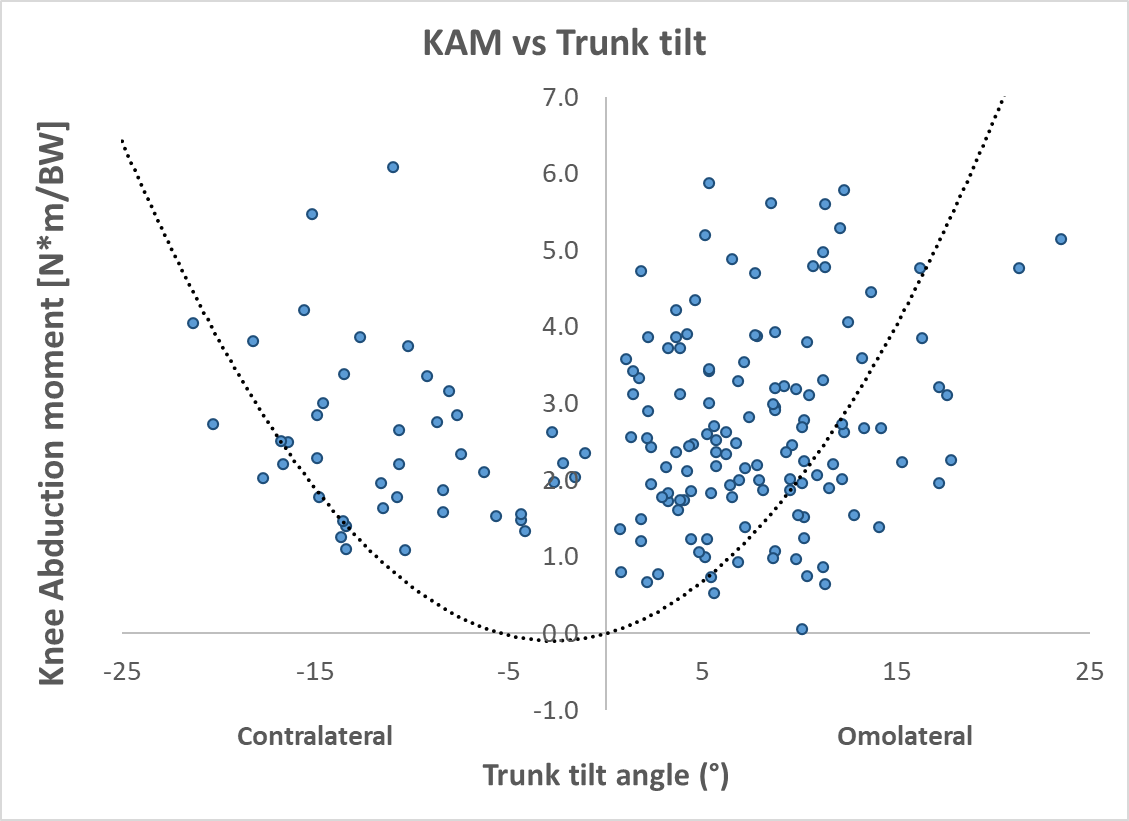


*Knee Abduction Moment (KAM, [N*m/BW]) evaluated through the 3D motion capture over the trunk tilt angle (°) evaluated through the 2D video analysis. A trend of increase in KAM can be noted as absolute trunk tilt angle increases.*
